# Extracellular vesicles derived from differentiated granulosa-like cells restore the ovarian function of rats with premature ovarian insufficiency

**DOI:** 10.1093/stcltm/szaf081

**Published:** 2026-01-26

**Authors:** Cheng Zou, Zelan Yang, Yan Zou, Hanyu Xiao, Yufei Deng, Jin Bai, Liaoqiong Fang, Zhibiao Wang

**Affiliations:** State Key Laboratory of Ultrasound in Medicine and Engineering, College of Biomedical Engineering, Chongqing Medical University, Chongqing 400016, China; State Key Laboratory of Ultrasound in Medicine and Engineering, College of Biomedical Engineering, Chongqing Medical University, Chongqing 400016, China; State Key Laboratory of Ultrasound in Medicine and Engineering, College of Biomedical Engineering, Chongqing Medical University, Chongqing 400016, China; State Key Laboratory of Ultrasound in Medicine and Engineering, College of Biomedical Engineering, Chongqing Medical University, Chongqing 400016, China; State Key Laboratory of Ultrasound in Medicine and Engineering, College of Biomedical Engineering, Chongqing Medical University, Chongqing 400016, China; State Key Laboratory of Ultrasound in Medicine and Engineering, College of Biomedical Engineering, Chongqing Medical University, Chongqing 400016, China; State Key Laboratory of Ultrasound in Medicine and Engineering, College of Biomedical Engineering, Chongqing Medical University, Chongqing 400016, China; National Engineering Research Center of Ultrasound Medicine, Chongqing 401121, China; State Key Laboratory of Ultrasound in Medicine and Engineering, College of Biomedical Engineering, Chongqing Medical University, Chongqing 400016, China

**Keywords:** premature ovarian insufficiency (POI), ovarian granulosa cell (OGC), umbilical cord mesenchymal stem cell (UCMSC), differentiation, granulosa-like cell

## Abstract

Specifically differentiated cells exhibit greater therapeutic efficacy than mesenchymal stem cells (MSCs), and extracellular vesicles (EVs) present therapeutic benefits similar to those of parental cells and fewer safety issues. Ovarian granulosa cells (OGCs) play a critical role in the pathogenesis of premature ovarian insufficiency (POI), a common gynecological disease that can cause infertility and has no effective treatment. Here, we investigated whether umbilical cord mesenchymal stem cells (UCMSCs) can differentiate into ovarian granulosa-like cells (GLCs) and whether GLC-EVs are more effective in restoring ovarian function than UCMSC-EVs are in POI model rats. Here, we differentiated rat UCMSCs (rUCMSCs) into GLCs in vitro using cytokines and hormones and isolated GLC-EVs. We then used chemotherapy-induced POI model rats to verify the ability of GLC-EVs to repair ovarian function. We found that GLCs/GLCs-EVs expressed granulosa cell markers (FOXL2 and FSHR). We demonstrated that GLC-EVs outperformed rUCMSC-EVs by restoring the estrous cycle and ovarian morphology, increasing the number of follicles, regulating serum hormone levels, and restoring fertility in POI model rats. Mechanistically, GLC-EVs showed enhanced ovarian tropism. Proteomic analysis identified PLAU as a key component of GLC-EVs, and subsequent antibody blockade experiments demonstrated that PLAU contributes to primordial follicle activation through promoting FOXO3A phosphorylation (pFOXO3A). This study provides the first proof that EVs derived from differentiated cells enhance therapeutic precision for POI, improve the tissue targeting of EV therapy, and provide a generalized strategy for clinical cell-free therapy.

## Graphical abstract

**Graphical Abstract:**
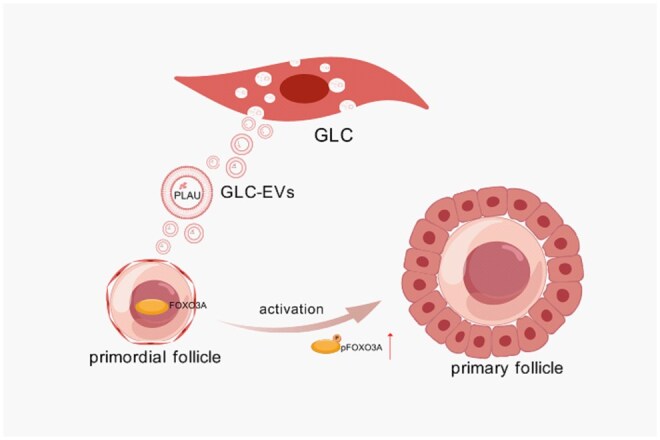
GLC-EVs highly express PLAU protein, which promotes the expression of pFOXO3A in the ovary and activates primordial follicles.

Significance statement We demonstrated that granulosa-like cell-derived EVs (GLC-EVs) outperform original UCMSC-EVs in restoring ovarian function in POI rats, effectively reactivating primordial follicles and recovering fertility. Notably, GLC-EVs demonstrate superior ovarian targeting and promote PLAU-associated FOXO3A phosphorylation, which correlates with primordial follicle activation. This differentiation-based EV engineering strategy provides a novel cell-free approach for ovarian dysfunction treatment, with potential application prospects.

## Introduction

Premature ovarian insufficiency (POI), a common gynecological disease, is the primary cause of infertility in women under the age of 40 years.^1^ The main manifestations are amenorrhea with increased FSH levels before the age of 40 years and a decreased number of follicles.^2^ At present, effective treatments to protect and restore ovarian function, which severely affects female reproductive health, are lacking.

Ovarian granulosa cells (OGCs) are the core components of follicular development and ovarian function and play a key role in follicular homeostasis by regulating oocyte growth, maturation, and hormone secretion. The meticulous regulation of folliculogenesis is crucial for female fertility in mammals. The primordial follicles, which constitute the finite ovarian reserve, are composed of a single oocyte and a layer of squamous pregranulosa cells (pre-GCs) that are precursors to granulosa cells (GCs).^3^ GCs are key somatic components of the ovary. They are critical for follicular development by supporting oocyte growth and proliferation and by producing essential sex steroids and various growth factors.^4^ The activation of a primordial follicle initiates with the oocyte achieving a diameter greater than 20 μm along with the proliferation and morphological transition of squamous pre-GCs into cuboidal/columnar granulosa cells.^5^ The in vitro activation of dormant primordial follicles has been established as a therapeutic strategy for patients with POI.^6^

MSCs, including UCMSCs,^7^ have been shown to have a therapeutic effect on POI,^8–10^ but their application is limited by their uncontrollable differentiation^11^ and degeneration and loss of stemness with aging in vivo and in vitro.^12^ Therefore, many researchers have begun to explore the effectiveness of other MSC strategies, such as the targeted differentiation of MSCs, in the treatment of diseases. Because a particular type of cells differentiated from stem cells contain proteins and molecules that are unique to that particular cell type, these molecules may have a more direct therapeutic effect on a particular disease or injury. For example, induced-differentiated hepatocellular-like cells^13–19^ and neural-like cells^20^ demonstrate promising potential in liver transplantation and the treatment of nerve injury. Furthermore, granulosa-like cells induced from MSCs in vitro exhibit characteristics of steroidogenesis and promotion of follicle formation.^21–23^ In addition, transplantation of induced granulosa-like cells could restore cyclophosphamide-induced ovarian injury and improve ovarian function,^24^ providing another possibility for the treatment of POI. In our preliminary research, we have successfully induced UCMSCs to differentiate into granulosa-like cells,^25^ but further research on the therapeutic effects of these induced granulosa-like cells is still pending.

Extracellular vesicles (EVs), which are low-toxicity, low-immunogenicity, biodegradable, and biocompatible, transport intracellular cargo locally and across long distances, mirror the distinctive features of secretory cells, and foster cell-specific connections through their specific surface proteins and receptors.^26^^,^^27^ Owing to the development of rapid methods for isolation, purification, and identification, EVs have emerged as potential agents for various medical applications. Mesenchymal stem cell–derived extracellular vesicles (MSC-EVs) represent a promising therapeutic strategy for the management of reproductive disorders, not only because they are derived from parental cells but also because of their greater biological stability and lower immunogenicity than MSCs.^28^ Evidence from previous studies indicates that MSC-derived EVs are capable of restoring ovarian function in premature senescent ovaries.^29^^,^^30^ However, under the premise that MSCs are capable of directed differentiation into specific cells, it is still unclear whether the EVs isolated from specific cells differentiated from MSCs have a more direct therapeutic effect on specific diseases or injuries than their parental cells do. Moreover, whether EVs generated from UCMSC-differentiated GLCs can also restore the function of premature senescent ovaries has not been demonstrated.

FOXO3, a key transcription factor in the FOXO family, plays critical roles in ovarian function. Its nuclear localization induces cell cycle arrest and apoptosis in oocytes, while its export and phosphorylation in the cytoplasm promote oocyte survival.^31–33^ FOXO3A regulates the activation of primordial follicles by translocating from the nucleus to the cytoplasm and undergoing phosphorylation.^34^ Female mice with FOXO3A knockout show global primordial follicle activation, oocyte loss, follicular depletion, and eventual infertility or ovarian insufficiency.^35^ Conversely, oocyte-specific nuclear overexpression of FOXO3 enhances reproductive performance, increases follicle numbers, and improves ovarian reserve.^36^ Additionally, FOXO3 dysregulation may contribute to granulosa cell apoptosis and impaired follicular development.^37^

The most optimal and readily accessible source of MSCs is likely the umbilical cord because of their noninvasive and easy extraction, high proliferative capacity, and potent immunomodulatory properties compared with those collected from other sites in the body.^38^ Umbilical cord–derived MSCs (UCMSCs) can differentiate into germ-like cells, indicating potential therapeutic value for infertility.^7^ However, there are no reports on the directional differentiation of UCMSCs into OGCs. Our study demonstrates that differentiation of UCMSCs into GLCs yields therapeutic extracellular vesicles capable of specifically restoring rat ovarian function. Furthermore, we have elucidated the mechanism by which its functional role is related to urokinase-type plasminogen activator (PLAU) promoting FOXO3A phosphorylation.

## Materials and methods

### Ethical approval

The animal experiment procedures involved in this study strictly follow the principles of animal protection, animal welfare and ethics, and have been approved by Institutional Animal Care and Use of Chongqing Medical University (IACUC-CQMU-2024-0869).

### Materials and animals

The rats were allowed to acclimatize for 1 week. Each rat was placed in an IVC cage (23°C ± 2 °C, relative humidity 55% ± 5%) under a 12-h light/dark cycle. Supplementary Table S1 lists the qPCR primer names, gene names, forward and reverse primer sequences, and gene accession numbers. The primers were synthesized by Tsingke Biotech Company. Supplementary Table S2 lists the primary and secondary antibodies used for immunofluorescence, immunohistochemistry, Western blotting, and flow cytometric analysis.

### Isolation and identification of rUCMSCs

Eight-week-old female Sprague–Dawley (SD) rats were housed with 10–12 weeks male stud rats for mating until pregnancy was confirmed. UCMSCs were derived from the umbilical cords of fetuses obtained from pregnant SD rats at 18–21 days of gestation. The rats were sacrificed on Day 18 postconception. The umbilical cords were collected under aseptic conditions, the amniotic membrane and blood were removed, the samples were cut into approximately 1 mm^3^ volume blocks, and digested with 1 mg/mL collagenase IV (C8160; Solarbio) to obtain rUCMSCs. These rUCMSCs were resuspended in DMEM/F12 (Cat. No. 6124281; Gibco), which was supplemented with 10% fetal bovine serum (JC65986; Clark) and 1% penicillin/streptomycin (BL505A; Biosharp), and cultured in a 75 cm^2^ T-flask under incubation conditions (37 °C and 5% CO_2_). After confluency reached approximately 90%, the adherent cells were digested with 0.25% trypsin/EDTA (C25200056; Gibco), collected by centrifugation, and subcultured at a ratio of 1:2. The MSCs were identified through morphology and characterization of MSC proteins. We confirmed their morphology by using a light microscope (OLYMPUS, Japan). Flow cytometry and immunofluorescence were used to detect MSC-specific surface markers (CD34, CD44, CD45, CD29, and CD90).

### Differentiation of rUCMSCs into granulosa-like cells

rUCMSCs were extracted from the rat umbilical cord. Passage 3 rUCMSCs were used for differentiation and cultured in a humidified incubator with 5% CO_2_ at 37 °C. rUCMSCs were cultured in DMEM/F12 supplemented with 10% FBS for 2 days. After 2 days, when the degree of cell aggregation reached 80%, the cell medium was replaced with differentiation medium for subsequent induction into GLCs. Estradiol (E2), transforming growth factor‑β (TGF‑β), and follicle‑stimulating hormone (FSH) were used for differentiation according to a previous protocol.^25^ The differentiation medium used for granulosa-like cells consisted of DMEM/F12, 10% FBS, 20 ng/mL E2 (E2758; Sigma), 50 ng/mL FSH (HY-P74133; MCE), and 15 ng/mL TGF-β (HY-P7118; MCE). The rUCMSCs were differentiated and cultured for 24 days, and the medium was changed every 2 days. The GLCs were identified based on their morphology, OGCs specific markers, and steroidogenesis capacity. We confirmed their morphology by using a light microscope (OLYMPUS, Japan). The expression of the pluripotency gene *Oct4* and granulosa cell markers (*Foxl2, Amhr2, Cyp19a1, Fshr*) was detected by immunofluorescence and PCR, while ELISA was used to measure E2 and anti-Mullerian hormone (AMH) levels in the culture supernatant of GLCs.

### Isolation and identification of EVs

When the P3 rUCMSCs and GLCs reached 80% confluence, the complete medium was replaced with serum-free DMEM/F12 basal medium. After 48 h of culture, the conditioned supernatant was collected. It was first centrifuged at 500×*g* for 15 min, followed by 2000×*g* for 15 min to remove cells and large debris. The supernatant was then passed through a 1.2-μm filter to eliminate remaining debris. Finally, EVs were isolated by two cycles of ultracentrifugation at 100 000×*g* for 70 min each. The precipitate was subsequently suspended in precooled PBS. The rUCMSC-EVs and GLC-EVs were experimentally identified. First, we used a Zetaview-PMX120-Z instrument (Particle Metrix, Germany) to analyse EV size distribution and concentration. Then, we used transmission electron microscopy (TEM; G2 12; FEI TECNAI, US) to observe their morphologies. Finally, characteristic proteins of EVs, including CD63, TSG101, and CD81 (BioLegend, US), negative proteins of EVs, and calnexin, were detected by western blotting. The rUCMSC-EV-related pluripotency protein OCT4 (Cat. No. SC-5279; Mouse; Santa Cruz), the GLC-EV-related protein FOXL2 (Cat. No. A16244; Rabbit; ABclonal), and FSHR (Cat. No. A1480; Rabbit; ABclonal) were also detected.

### Establishment and treatment of POI model rats

Female SD rats (6–8 weeks old) were purchased from the Laboratory Animal Center of Chongqing Medical University. Rats with regular estrous cycles, as determined by vaginal smear, were selected for the study. To induce POI, the rats received an initial intraperitoneal injection of cyclophosphamide (120 mg/kg) and were then maintained on a daily dose of 8 mg/kg for the following 13 days (C0768, Sigma, Germany). Vaginal smears were performed daily over the entire 28-day duration of the animal experiment. After POI model established, 100 model rats were randomly divided into three groups, the control group (100 µL PBS), the rUCMSC-EVs group (50 μg in 100 µL PBS), and the GLC-EVs group (50 μg in 100 µL PBS). All groups received their respective treatments via intraperitoneal injection once per week for a total of two sessions. The injection dose of EVs was determined based on previous studies.^39^ Sixteen healthy female SD rats were assigned to the normal group. For blood collection, the animals were anesthetized with 2% isoflurane, and blood was collected through cardiac puncture. Blood sample was centrifuged at 1000×*g* for 5 min to obtain the serum. Finally, the animals were euthanized for the collection of organs, including the brain, heart, lung, liver, spleen, kidney, uterus, and ovary. All animals were euthanized by 2% isoflurane inhalation anesthesia and cervical dislocation before tissue collection. After the excision of adjacent adipose tissue and fallopian tubes, the weights of the ovaries and the ratios of ovary weight to body weight were assessed (ovary index = ovary weight/body weight × 100%). One ovary was subsequently prepared for histological examination, while the other ovary and serum samples were preserved at −80 °C.

### Reproductive tests

After POI model rats were treated with EVs for 2 weeks, eight rats per group were randomly chosen and mated with 10–12 weeks healthy male stud SD rats in a 1:2 (female: male) pairing. Successful mating was verified by the detection of a vaginal sperm plug the next morning.

### Ovarian follicle counts and morphological analysis

We fixed the collected ovaries in 4% paraformaldehyde (BL539A; Biosharp) for 48 h. And the ovaries were dehydrated, embedded in paraffin, and cut into 4-μm serial sections. For the largest cross section of the ovary, five slices with a thickness of 40 μm were serially sectioned, one of which was used for follicle counting. Five sections from each ovary were selected for hematoxylin–eosin (HE) staining. Finally, we scanned and observed the morphological characteristics of every ovarian section. Follicles at all developmental stages, including primordial, primary, secondary, and antral, were enumerated using the K-Viewer system (KFBIO, China).

### EV tracing

EVs were labeled with 5 µmol/mL DiR (HY-D1048; MCE) for intraperitoneal injection in POI model rats, and the main organs (brain, heart, lung, liver, kidney, spleen, uterus, and ovary) were harvested and used for bioluminescence imaging (BLI) 48 h later using an in vivo animal imaging system IndiGo (BERTHOLD, Germany).

### Elisa

Levels of AMH and E_2_ (in serum and culture supernatant) and serum FSH were analyzed using ELISA kits (Aidisheng Biological Technology Co. Ltd, Jiangsu). In short, samples were incubated in the wells of a 96-well plate pre-coated with peroxidase-conjugated antibodies against AMH, E2, or FSH. Following the enzyme reaction, the measurement was carried out at 450 nm with a microplate reader (Tecan, Switzerland).

### In vitro ovarian follicle count determination and immunohistochemistry

The ovaries of 2.5-day-old female SD pups were cultured in complete DMEM/F12 supplemented with 10% FBS in vitro. Those ovaries were divided into four groups: the complete medium group (control), the GLC-EVs group, the GLC-EVs + anti-PLAU group, and the anti-PLAU group, with three ovaries in each group. For the GLC-EVs group, 2 µg/mL GLC-EVs were added, for the GLC-EVs+ anti-PLAU group, 2 µg/mL GLC-EVs and 1 ng/mL anti-PLAU antibody (Cat. No. A2181; Rabbit; ABclonal) were added 2 h prior, and for the anti-PLAU group, 1 ng/mL anti-PLAU antibody was added. The incubation period was 6 days, and the medium was changed every other day. After in vitro ovarian culture, HE staining was used for primary follicle counting, and immunohistochemistry was used for primitive follicle-activating protein (FOXO3A, phospho-FOXO3A) staining.

### Detection of extracellular vesicle contents

In this study, mass spectrometry–based proteomics technology was used to identify and quantify proteins in two kinds of vesicles, namely rUCMSC-EVs and GLC-EVs. The raw mass spectrometry data were processed by Spectronaut software (version 19) for protein identification using the rat UniProt database (version 2023.11). The false-positive rate (FDR) for peptides and proteins was set at 1%. Label-free quantification (LFQ) was used for protein quantification, and experimental error was eliminated by normalization. The screening criteria for differentially expressed proteins were as follows: |log2(fold change)| ≥ 1 and *P* value < 0.05 (based on a *t*-test). To further analyze the functional characteristics of the differentially expressed proteins, Gene Ontology (GO) functional annotation and Kyoto Encyclopedia of Genes and Genomes (KEGG) pathway enrichment analysis were performed using GO tools (version 1.4.4). The threshold for significance was set at a *P* value < 0.05. The data were analyzed on the online platform of Majorbio Cloud Platform (www.majorbio.com).

### Real-time PCR

Extraction of total RNA was performed with TRIzol Reagent (Cat. No. 98597501; Invitrogen). The RNA concentration was subsequently determined using a NanoDrop spectrophotometer (Thermo Fisher Scientific). For cDNA synthesis, total RNA was subjected to reverse transcription using a reverse transcription kit (Cat. No. R323-01; Vazyme) on a SureCycler 8800 thermocycler (Agilent). Quantitative real-time PCR (qPCR) was performed on the cDNA template with a SYBR Green qPCR kit (Cat. No. 1129280; QIAGEN) and an AriaMx real-time PCR system (Agilent). Gene expression was normalized to *Gapdh*, and relative mRNA levels were calculated using the ΔΔC_T_ method.

### Immunofluorescence

After fixation with 4% paraformaldehyde and permeabilization with 0.1% Triton X-100 (Cat. No. T8200; Solarbio), cells were blocked with QuickBlock™ immunostaining solution (Cat. No. P0260; Beyotime). And then cells were incubated with the primary antibodies overnight at 4 °C. The primary antibodies included FOXL2 (Cat. No. A16244; Rabbit; ABclonal), OCT4 (Cat. No. SC-5279; Mouse; Santa Cruz), AMHR2 (Cat. No. SC‐377413; Mouse; Santa Cruz), CYP19A1 (Cat. No. SC-374176; Mouse; Santa Cruz), FSHR (Cat. No. A1480; Rabbit; ABclonal), CD29-PE (Cat. No.102207; Mouse; BioLegend), CD90-FITC (Cat. No. 206105; Mouse; BioLegend), CD45-PE (Cat. No. 202207; Mouse; BioLegend), CD34 (Cat. No. SC-7324; Mouse; Santa Cruz), and CD44 (Cat. No. SC-7297; Mouse; Santa Cruz). For detection, the cells were incubated with species-appropriate fluorescent secondary antibodies for 1 h in the dark, including Alexa Fluor 488 rabbit anti-mouse IgG (Cat. No. 33906ES60; Yeasen), Alexa Fluor 488 donkey anti-rabbit IgG (Cat. No. 34206ES60; Yeasen), Alexa Fluor 594 rabbit anti-mouse IgG (Cat. No. 33912ES60; Yeasen), and Alexa Fluor 594 donkey anti-rabbit IgG (Cat. No. 34212ES60; Yeasen). Finally, the cells were stained with DAPI (Beyotime; Cat. No. C1006) and mounted with Echo Revolve (ECHO, USA).

### Flow cytometric analysis

The cells were trypsinized with trypsin/EDTA solution (Cat. No. 2509042; Gibco). For staining, the following antibodies were applied: anti-FOXL2 (Cat. No. A16244; Rabbit; ABclonal), anti-OCT4 (Cat. No. SC-5279; Mouse; Santa Cruz), anti-CD29-PE (Cat. No. 102207; Mouse; BioLegend), anti-CD90-FITC (Cat. No. 206105; Mouse; BioLegend), anti-CD45-PE (Cat. No. 202207; Mouse; BioLegend), anti-CD34 (Cat. No. SC-7324; Mouse; Santa Cruz), and anti-CD44 (Cat. No. SC-7297; Mouse; Santa Cruz). Prior to primary antibody staining, the cells were fixed, permeabilized, and blocked in PBS supplemented with 5% fetal bovine serum. Isotype control cells were incubated with nonspecific isotype-matched antibodies. For detection, the secondary antibodies used included FITC-labeled donkey anti-mouse IgG (Cat. No. 33906ES60; Yeasen) or anti-rabbit IgG (Cat. No. 34206ES60; Yeasen) and PE-labeled donkey anti-rabbit IgG (Cat. No. 34212ES60; Yeasen) or anti-mouse IgG (Cat. No. 33912ES60; Yeasen). The stained single-cell suspensions were analyzed using a CytoFLEX flow cytometer (Beckman Coulter).

### Western blotting

Ovarian proteins were extracted using RIPA lysis buffer (Beyotime). Protein concentrations were quantified with a BCA assay kit (Beyotime), and samples were subsequently denatured by heating at 100 °C for 10 min. The proteins were separated by SDS-PAGE (Beyotime) and transferred onto 0.22 μm PVDF membranes (Millipore). The membranes were blocked in 5% bovine serum albumin (BSA) for 2 h at room temperature and then incubated with primary antibodies overnight at 4 °C. The next day, the membranes were incubated for 1 h with HRP-conjugated secondary antibodies: goat anti-rabbit (Cat. No. GAR007; MultiSciences) and goat anti-mouse (Cat. No. GAM007; MultiSciences). Finally, protein levels were detected using the cSeries capture software (Azure Biosystems, USA).

### Immunohistochemistry

Immunohistochemistry was used to identify activated primordial follicles. Two biomarkers, FOXO3A (Cat. No. A0102; Rabbit; ABclonal) and phospho-FOXO3A (Cat. No. AP0892; Rabbit; ABclonal), were selected for primordial follicle activation. For immunohistochemistry, the following procedures were used: antigen recovery, peroxidase blocking, primary antibody incubation, secondary antibody ligation, diaminobenzidine staining, and Mayer hematoxylin counterstaining. Brown cytoplasmic staining indicates activated primordial follicles.

### Statistical analysis

The statistical analyses were performed via ordinary one-way ANOVA and unpaired two-tailed *t*-tests, followed by post hoc Dunnett’s multiple comparisons test. All data from at least three independent measurements are presented as the means ± SEMs. Statistical analyses were performed using GraphPad Prism version 8.0 (GraphPad, San Diego, CA). *P* values <0.05, 0.01, 0.001, and 0.0001 were considered statistically significant.

## Results

### Characterization of GLC-EVs and rUCMSC-EVs

**Figure 1.**
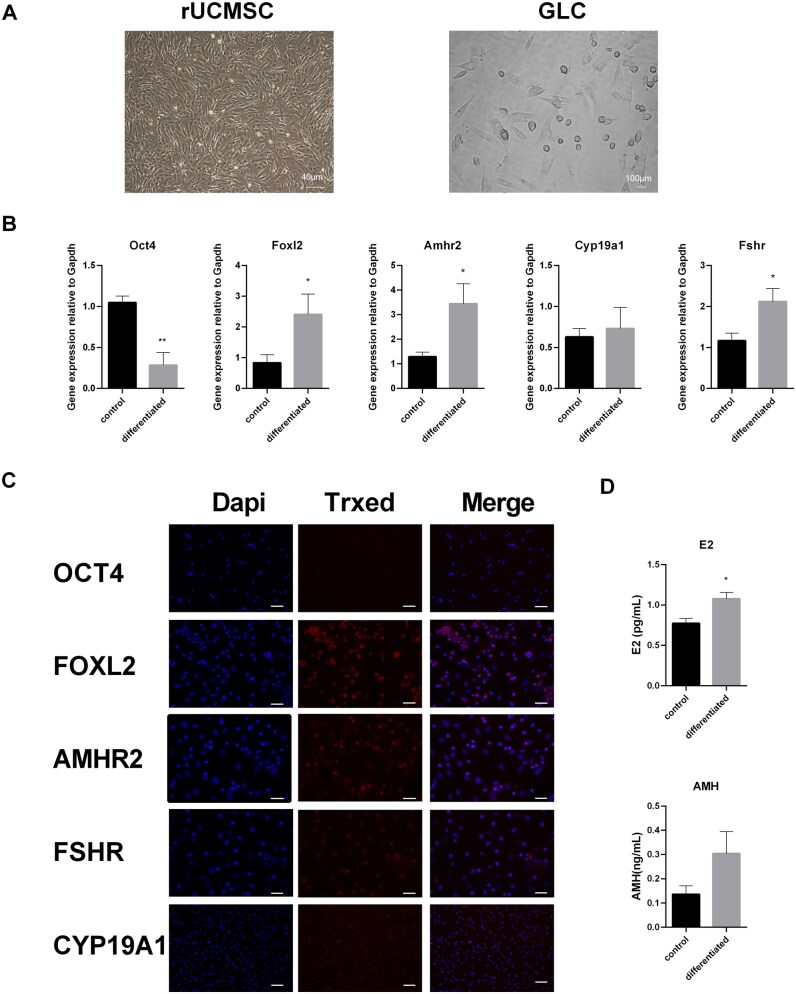
Granulosa cell marker proteins in rUCMSC-derived granulosa-like cells. (A) rUCMSC and GLC cell morphology. (B) Real-time PCR was used to analyze the gene expression levels of the stem cell pluripotency marker Oct4; the specific precursor granulosa cell marker *Foxl2;* and the mature granulosa cell markers *Amhr2*, *Cyp19a1*, and *Fshr* during the differentiation of rUCMSCs into granulosa cells. The values are the means ± standard errors of the means (SEMs) (*n* = 5). The control (Crl) cells were undifferentiated rUCMSCs (Day 0). *Gapdh* was used as an internal control for quantification. *P < 0.05; **P < 0.01. (C) Immunofluorescence images of granulosa cell marker proteins and stem cell pluripotency marker proteins. Bar: 100 µm. (D) AMH and E2 hormone secretion level of differentiated and undifferentiated rUCMSC in vitro (*n* = 3). Control (Crl) cells are undifferentiated rUCMSCs. *P < 0.05.

The isolated rUCMSCs were displayed a long-spindle, fibroblast-like morphology and a surface marker profile positive for CD44, CD29, and CD90 and negative for CD34 and CD45 (Figure S1A–C). GLCs formed by rUCMSC differentiation were short spindle-shaped or round and tested positive for FOXL2, AMHR2, CYP19A1, and FSHR and negative for OCT4 (Figure 1A–C). Furthermore, after differentiation, the GLCs showed an increased capacity to produce E2 and AMH compared to the undifferentiated state (Figure 1D).

**Figure 2.**
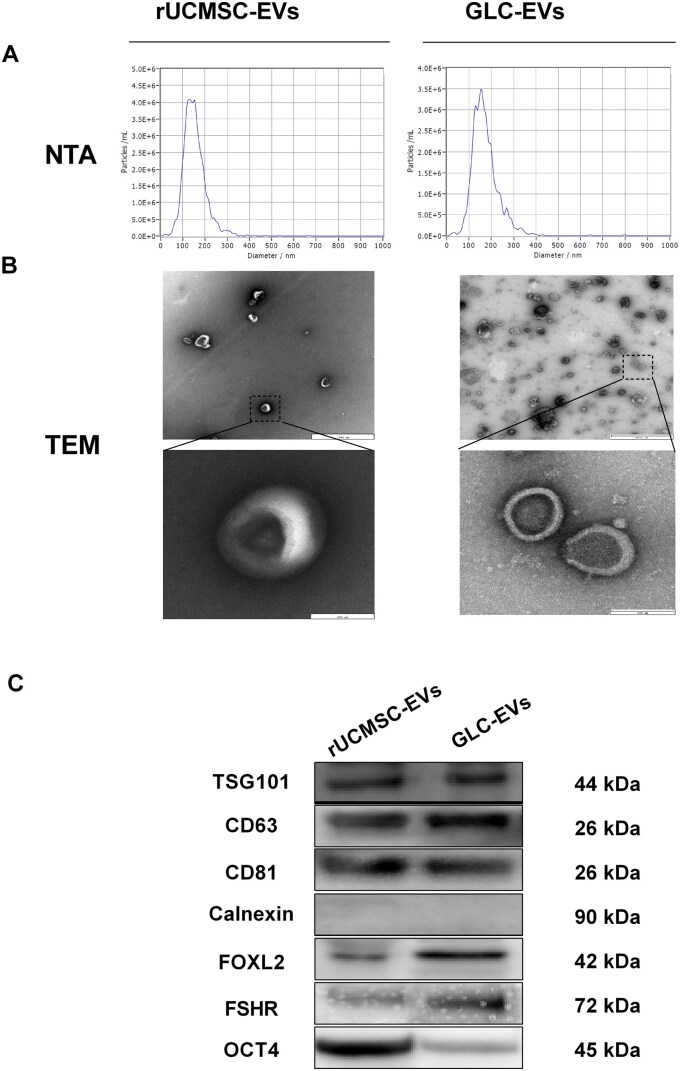
EV characterization. (A) Nanoparticle tracking analysis of the particle size distribution and concentration of rUCMSC-EVs and GLC-EVs. (B) Representative images of rUCMSC-EVs and GLC-EVs observed by transmission electron microscopy (TEM). Scale bar: 200 nm. (C) WB analysis of EV-associated markers (Tsg101, CD63, and CD81), GLC-EV markers (FOXL2 and FSHR), and an MSC pluripotency marker (OCT4). The cellular endoplasmic reticulum protein calnexin was used as a negative control.

GLC-EVs and rUCMSC-EVs were isolated from serum-free culture supernatants and subjected to standard characterization. NTA revealed that GLC-EVs and rUCMSC-EVs had concentrations of 7.4 × 10^10^ particles/mL and 4.9 × 10^10^ particles/mL, with peak diameters of 128.9 and 122.8 nm, respectively (Figure 2A). TEM confirmed a sphere-shaped morphology in both GLC-EVs and rUCMSC-EVs (Figure 2B). BCA analysis demonstrated that the protein concentrations of the GLC-EVs and rUCMSC-EVs were 0.86 and 0.96 mg/mL, respectively. Western blot analysis confirmed the positive expression of the EV-specific markers CD63, TSG101, and CD81 and the absence of the negative marker calnexin. GLC-EVs positively expressed the granulosa cell characteristic proteins FOXL2 and FSHR but negatively expressed the stem cell pluripotency characteristic protein OCT4 according to western blot analysis, whereas rUCMSC-EVs were negative for FOXL2 and FSHR but positive for OCT4 (Figure 2C).

### GLC-EVs and rUCMSC-EVs treatment restored the estrous cycle and ovarian morphology in POI model rats in vivo

**Figure 3.**
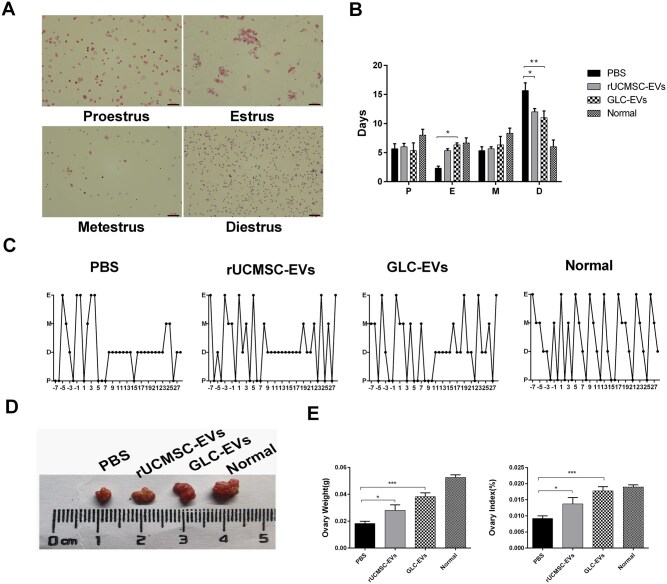
Rat estrous cycle and ovarian morphology. (A) HE staining of vaginal smears from normal rat estrous cycles. Scale bar: 100 µm. (B) Number of mice in each stage of the estrous cycle in different groups (*n* = 3; P, proestrus; E, estrus; M, metestrus; D, diestrus). *P < 0.05, **P < 0.01. (C) Typical estrous cycle patterns in normal rats and rats in the three treatment groups. (D) Ovarian morphology in each group. (E) Ovary weight (left) and the ovary/body weight ratio (right) (*n* = 4). *P < 0.05, ***P < 0.001.

To monitor and compare the estrous cycles across different groups, vaginal smear was performed. Normal rats maintained regular, stable estrous cycles. The PBS group was predominantly in diestrus (D), with subsequent metestrus (M) and proestrus (P) stages and a near-total absence of estrus (E). Administration of GLC-EVs significantly increased the occurrence of the E stage. A significant reduction in the D stage was also observed in both rUCMSC-EVs and GLC-EVs groups compared to the PBS group (Figure 3A–C).

Although cyclophosphamide injection decreased the ovarian size, this effect was improved after receiving rUCMSC-EVs or GLC-EVs transplantation (Figure 3D). Compared with those in PBS group, the ovarian weight and ovarian index were significantly greater in both the rUCMSC-EV and GLC-EV groups, especially in the GLC-EVs group (Figure 3E).

### GLC-EVs targeted the ovaries of POI model rats

**Figure 4.**
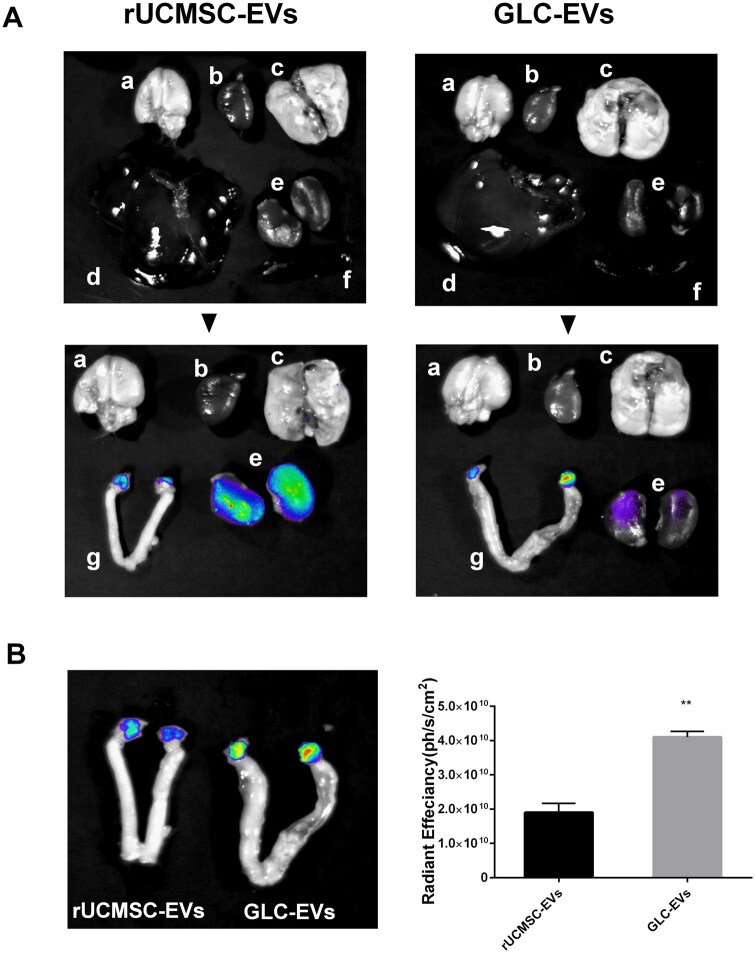
Ovarian tropism of rUCMSC-EVs and GLC-EVs. (A) DiR fluorescence intensity of major organs. (a) Brain, (b) heart, (c) lung, (d) liver, (e) kidney, (f) spleen, and (g) uterus and ovary. Blue, low fluorescence intensity; green, medium fluorescence intensity; red, high fluorescence intensity. (B) Comparison of fluorescence intensity in the ovaries of rUCMSC-EV- and GLC-EV-treated rats (*n* = 3). **P < 0.01.

For in vivo tracking, the transplanted EVs were labeled with DiR. BLI revealed a distinct biodistribution pattern for each group. In the rUCMSC-EVs group, the highest DiR intensity was localized to the kidneys, with a weaker signal in the ovaries. Conversely, in the GLC-EVs group, the strongest fluorescence was observed in the ovaries, followed by a weaker renal signal. No DiR signal was detectable in the brain, heart, lung, liver, spleen, or uterus in either group (Figure 4A). A comparison of DiR fluorescence in the ovaries of the rUCMSC-EV and GLC-EV groups revealed that DiR fluorescence was markedly stronger in the GLC-EVs group than in the rUCMSC-EVs group (Figure 4B), suggesting that GLC-EVs have greater affinity to the ovaries.

### GLC-EVs were superior to rUCMSC-EVs in restoring ovarian structural morphology, promoted follicular development, regulated serum hormone levels, and protected fertility in a rat model of POI

**Figure 5.**
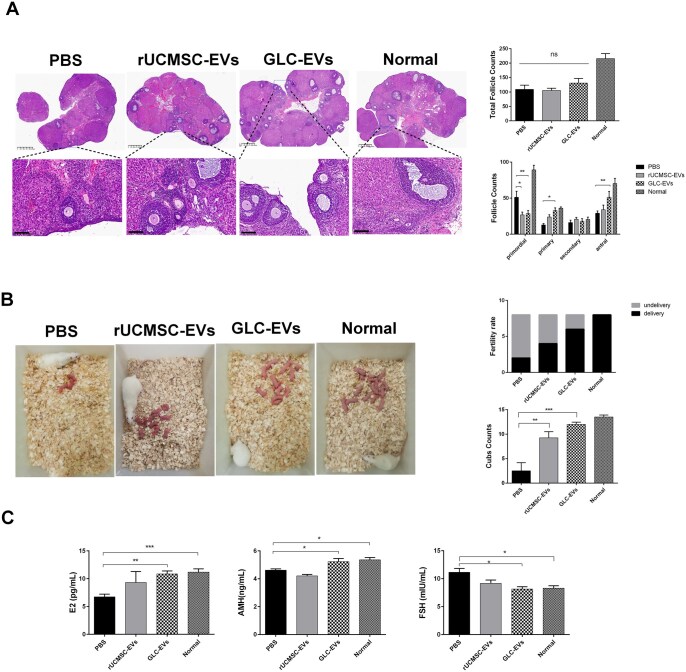
Ovarian histological morphology, ELISA results, and live birth outcomes after treatment. (A) H&E staining of ovarian sections, total follicle counts, and counts of follicles at different stages (*n* = 4). Scale bars: 625 μm for the main image and 100 μm for the inset. (B) Live birth images of normal rats and rats in the three treatment groups and live birth counts (*n* = 8). (C) Serum levels of E2 (left), AMH (middle), and FSH (right) (*n* = 3–4). The data are presented as the means ± SEM; *P < 0.05, **P < 0.01, ***P < 0.001.

Histological analyses demonstrated that intraperitoneal injection of cyclophosphamide caused ovarian atrophy. The total number of follicles revealed no significant differences among the three groups: PBS (109 ± 38.19), rUCMSC-EVs (105 ± 15.58), and GLC-EVs (130 ± 45.48) (Figure 5A). The count of primordial follicles was 51 ± 22.3 in the PBS group, which was markedly elevated compared to that in the rUCMSC-EVs (26 ± 7.76) and GLC-EVs (28.5 ± 11.99) groups. Regarding primary follicles, the number in the PBS group was 12 ± 5.11, while both the rUCMSC-EVs (23 ± 6.65) and GLC-EVs (32 ± 10.32) groups exhibited a significant increase. The secondary follicle counts were 16 ± 8.69 in the PBS, 20.75 ± 4.99 in the rUCMSC-EVs, and 18 ± 9.67 in the GLC-EVs group, with no statistically significant differences observed. For antral follicles, the PBS group had 29 ± 8.19, compared to 34 ± 12.57 in the rUCMSC-EVs and 51 ± 22.88 in the GLC-EVs group, a significant difference was detected between the PBS and GLC-EVs groups(Figure 5A). Histological analyses of important organs in the four groups revealed no abnormal changes in the heart, liver, spleen, lung, kidney, and brain. (Figure S2).

Fertility was assessed across treatment groups via mating experiments. Although the pregnancy rate showed an increasing trend in the three treatment groups compared to the PBS group, the difference was not statistically significant. However, based on the number of offspring, both the rUCMSC-EVs and GLC-EVs groups had a significantly higher number of live births than the PBS group (Figure 5B). Furthermore, the GLC-EVs group yielded more live births than the rUCMSC-EVs group.

We next assessed serum E2, AMH, and FSH levels. POI rats, compared with normal rats, displayed a characteristic hormonal imbalance, with decreased E2 and AMH and increased FSH. This imbalance was significantly reversed by GLC-EVs treatment, which raised E2 and AMH and lowered FSH. In contrast, the rUCMSC-EVs and PBS groups showed no significant differences in any of these hormones (Figure 5C).

These results collectively indicated that GLC-EVs treatment alleviated cyclophosphamide-induced ovarian atrophy, thereby restoring the estrous cycle and promoting follicular development. Furthermore, repeated administration of GLC-EVs restored female sex hormone levels and ultimately improved fertility in POI model rats.

### The PLAU protein in GLC-EVs is essential for restoring ovarian function in POI rats

**Figure 6.**
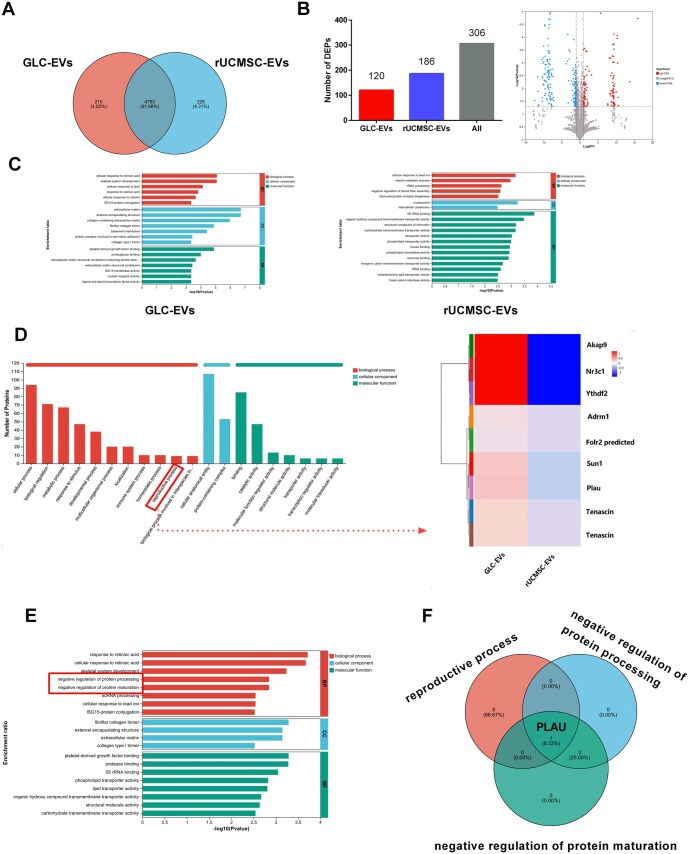
Analysis of the proteins contained in GLC-EVs and rUCMSC-EVs. (A) Venn diagram of GLC-EV and rUCMSC-EV proteins. (B) Bar plot and volcano plot of differentially expressed proteins between GLC-EVs and rUCMSC-EVs. The volcano plots show the differential expression of all identified proteins, where the significantly upregulated proteins are labeled in red (GLC-EVs) and blue (rUCMSC-EVs). (C) GLC-EVs and rUCMSC-EVs for GO analysis. (D) GO annotation analysis of GLC-EV vs rUCMSC-EV upregulated proteins and heatmap of proteins involved in reproductive processes. (E) GO enrichment analysis of differentially expressed proteins between GLC-EVs and rUCMSC-EVs. (F) Venn diagram of negative regulatory proteins involved in reproductive processes and protein processing/maturation.

The abundant proteins in EVs are instrumental in mediating their intracellular activity and intercellular communication.^40^ To delineate the underlying mechanism through which GLC-EVs treat premature ovarian failure in POI model rats, we used quantitative proteomics to determine the levels of different proteins in GLC-EV and rUCMSC-EV samples. A Venn diagram quantified 4993 and 5008 proteins in the GLC-EVs and rUCMSC-EVs groups, respectively (Figure 6A). Subsequent screening identified 306 differentially expressed proteins (DEPs) between them (*P* < 0.05, |log_2_ FC | > 1), with 120 upregulated and 186 downregulated (Figure 6B). Next, we explored the protein differences between GLC-EVs and rUCMSC-EVs. Gene Ontology (GO) analysis was performed to characterize the functional roles of the differentially expressed proteins in GLC-EVs versus rUCMSC-EVs. The results revealed that the upregulated proteins in the rUCMSC-EVs were involved mainly in metabolic processes and immune regulation, whereas the upregulated proteins in GLC-EVs were significantly enriched in biological processes including extracellular matrix organization, signal transduction, and cellular response to external stimuli (Figure 6C). GLC-EVs may have stronger tissue repair, metabolic regulation and stress response capabilities. We then performed a GO analysis to identify proteins involved in reproductive processes, and nine candidate proteins (*P* < 0.05, |log_2_ FC | > 1) were further screened using a heatmap (Figure 6D). Compared with those in rUCMSC-EVs, the proteins in GLC-EVs showed significant functional enrichment in the negative regulation of protein processing/maturation (Figure 6E). Protein processing/maturation is an important molecular mechanism that finely regulates protein function and activity to ensure normal ovarian physiological processes. Venn diagram analysis of the proteins involved in the negative regulation of protein processing/maturation and reproductive processes revealed PLAU as a shared protein (Figure 6F). PLAU/PLAUR-induced intracellular signals have been implicated in granulosa cell proliferation, informing the mechanisms governing early follicular development.^41^ These findings suggest that PLAU may be a candidate target protein.

### PLAU protein in GLC-EVs promotes primordial follicle activation

**Figure 7.**
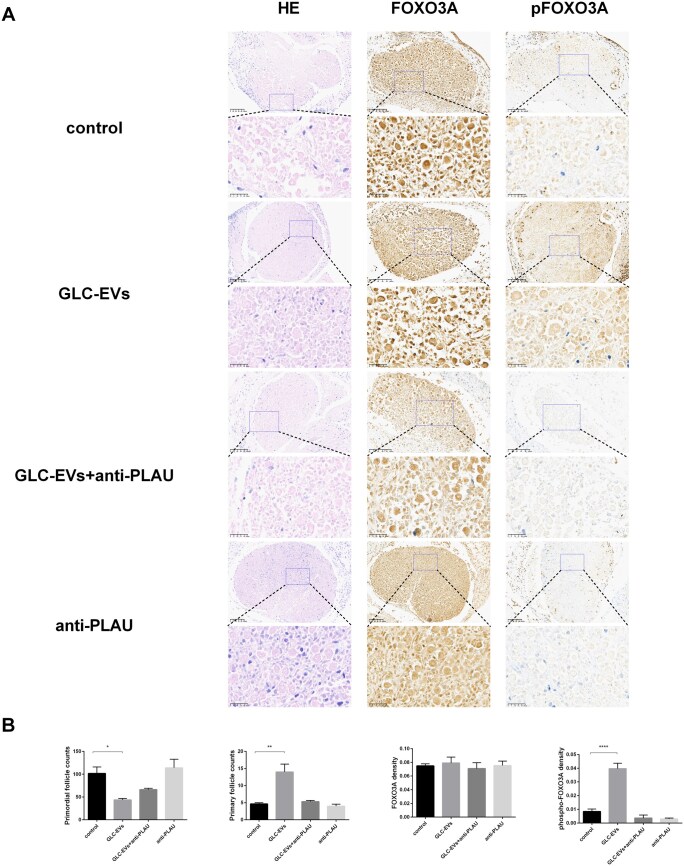
Protein staining and analysis of primordial follicle-activation in ovaries. (A) HE staining and immunohistochemistry of ovaries cultured in vitro. Scale bars: 100 μm for the main image and 25 μm for the inset. (B) Primordial follicle counts, primary ovarian follicle counts, and protein quantitation of densities of FOXO3A and pFOXO3A in vitro cultures (*n* = 3). *P < 0.05, **P < 0.01, and ****P < 0.0001 indicate a significant difference compared with the control.

Compared with the control group, the GLC-EVs group showed a marked decrease in primordial follicles (43.67 ± 3.18 vs 101.7 ± 14.24) but an increase in primary follicles (14 ± 2.31 vs 4.67 ± 0.33) (Figure 7B). Meanwhile, there was no significant difference between the GLC-EVs + anti-PLAU group and the anti-PLAU group in comparison with the control group. The semiquantitative immunohistochemical staining was applied to measure the FOXO3A and phospho-FOXO3A density in ovaries per section (Figure 7A). Indeed, statistical analysis revealed no significant differences in FOXO3A density across the four treatment groups, but strongly positive expression of pFOXO3A was detected in ovarian primordial follicles treated with GLC-EVs in vitro (Figure 7B), indicating significant activation of primordial follicles.

## Discussion

This study establishes a novel differentiation protocol to generate GLCs from UCMSCs and demonstrates that GLC-EVs exhibit superior therapeutic efficacy over naive MSC-EVs in POI. Our findings confirm that GLC-EVs reversed POI phenotypes, including primordial follicular activation, hormonal rebalancing, and fertility recovery and show enhanced ovarian tropism. This enhanced reparative effect is related to the promotion of phosphorylation of FOXO3A by PLAU to stimulate primordial follicle activation.

GCs are not only nurse cells that provide nutrient support to the oocyte but also possess endocrine functions, acting as key drivers of the reproductive process. Their differentiation and development play critical roles throughout the menstrual cycle and pregnancy and are closely linked to follicular fate.^42^ The quality of GCs directly impacts ovarian reserve and function, and GCs of inferior quality may ultimately lead to POI.^24^ GCs are a critical regulators of ovarian function and follicular maturation. Herein, we successfully directed the differentiation of rUCMSCs into GLCs using a combinatorial treatment of cell growth factors and hormones. The results showed that these rUCMSC-derived GLCs strongly expressed GC markers but no longer retained rUCMSC characteristics. Furthermore, the differentiated GLCs acquired the capacity to secrete steroid hormones. (Figure 1). Although encouraging results have been obtained, cell therapy still faces significant challenges, including tumorigenicity, low cell survival rates, and immune rejection.^43^ Therefore, rUCMSCs and GLC-derived EVs were extracted for comparative studies of restoring ovarian function in POI model rats. Comparative analysis of EV treatments revealed that GLC-EVs can reach the ovary more accurately (Figure 4), which demonstrated that EVs generated by differentiation into POI key cells were more ovarian targeted. Moreover, GLC-EVs significantly improve the therapeutic effect of targeting ovarian dysfunction in POI rats (Figures 3 and 5). This therapeutic superiority stems from GLC-EVs’ unique molecular signature, particularly their PLAU-enriched cargo.

PLAU, a key member of the plasminogen activation system, contributes significantly to follicular development and ovulation.^44^ PLAU produces plasminogen by activating plasminogen, which is involved in ovarian functions such as follicle rupture during ovulation.^45^ In addition, PLAU is involved in regulating granulosa cell function and oocyte maturation, thereby affecting follicle development and quality.^41^ We analyzed the differentially expressed proteins in rUCMSC-EVs and GLC-EVs by proteomics and found that PLAU was highly expressed in GLC-EVs and expressed at low levels in rUCMSC-EVs, which may be one of the important mechanisms by which GLC-EVs improve the ovarian function of POI model rats. Zhao’s research have suggested that PLAU is involved in extracellular matrix remodeling and cell migration, which are essential for tissue repair and tumor progression.^41^ In the ovary, PLAU is associated with follicle rupture during ovulation, and its role in granulosa cell proliferation has been suggested to involve PLAU interacting with PLAUR in the ovary to trigger cAMP signaling and activate the MAPK/ERK1/2 pathway, thereby promoting bovine GC proliferation.^41^ Our research has revealed that PLAU in GLC-EVs promoted the transition of ovarian follicles from the primordial to the primary stage (Figure 7). In vitro blockade of PLAU significantly attenuated phosphorylation of FOXO3A in primordial follicles compared to controls (Figure 7), confirming its pivotal role in ovarian functional recovery. These findings indicate that PLAU primarily regulates primordial follicle activation by promoting FOXO3A phosphorylation, providing a molecular basis for GLC-EVs’ superior therapeutic effects.

## Conclusion

To sum up, our study demonstrates that differentiation-derived granulosa-like cell EVs outperform original UCMSC-EVs in restoring ovarian function in POI. This suggests that directed differentiation of MSC can optimize the therapeutic targeting of EVs. This differentiation-based EV engineering strategy could have broader applications in regenerative medicine.

A limitation of the study is that while this study demonstrates the therapeutic potential of GLC-EVs, the precise molecular interplay between PLAU and FOXO3A phosphorylation warrants further validation in human cell models.

## Supplementary Material

szaf081_Supplementary_Data

## Data Availability

The data will be made available upon request.
