# Supplementary material for: Extracellular vesicles derived from differentiated granulosa-like cells restore the ovarian function of rats with premature ovarian insufficiency
Source: Stem Cells Transl Med. 2026 Jan 26;15(2):szaf081. doi: 10.1093/stcltm/szaf081 (PMC12832956; doi:10.1093/stcltm/szaf081)
Supplement: szaf081_Supplementary_Data [file szaf081_supplementary_data.docx]

**Supplementary Information**

**Extracellular Vesicles Derived** **from Differentiated Granulosa-like Cells Restore the Ovarian Function of Rats with Premature Ovarian Insufficiency**

Running title: GLC-EVs Restore Ovarian Function in POI

Cheng Zou ^1, a^, Zelan Yang ^1, a^, Yan Zou^1^, Hanyu Xiao^1^, Yufei Deng^1^, Jin Bai^1^, Liaoqiong Fang ^1, 2, *^, Zhibiao Wang ^1 *^

^1^ State Key Laboratory of Ultrasound in Medicine and Engineering, College of Biomedical Engineering, Chongqing Medical University, Chongqing, 400016, China.

^2^ National Engineering Research Center of Ultrasound Medicine, Chongqing, 401121, China.

*Correspondence should be addressed to: Prof. Zhibiao Wang, No.1 Yixueyuan Road, Yuzhong District, Chongqing Medical University, Chongqing, 400016, China. Tel: 13608335939. E-mail: [wangzb@cqmu.edu.cn](mailto:wangzb@cqmu.edu.cn); Prof. Liaoqiong Fang, No.1 Yixueyuan Road, Yuzhong District, Chongqing Medical University, Chongqing, 400016, China. Tel: 13650589665. E-mail: [lqfang06@163.com](mailto:lqfang06@163.com).

^a^ These authors contributed equally to this work.

**Supplementary Table S1. Primer information.**

| **Primer**  **Symbol** | **Gene name** | **Primer direction** | **Sequences (5’ to 3’)** | **Accession number** |
| --- | --- | --- | --- | --- |
| *Gapdh* | Glyceraldehyde 3-phosphate dehydrogenase | Forward | GAAGGTCGGTGTGAACGGAT | NM_017008 |
|  |  | Reverse | CCCATTTGATGTTAGCGGGAT |  |
| *Oct4* | POU class 5 homeobox 1 | Forward | CAGCTCAGCCTTAAGAACATGTG | NM_001009178 |
|  |  | Reverse | TTCTCAATGCTAGTCCGCTTTCT |  |
| *Foxl2* | forkhead box L2 | Forward | TCCGGCATCTACCAGTACATCAT | XM_003750571 |
|  |  | Reverse | GGTAGTTGCCCTTCTCGAACAT |  |
| *Amhr2* | anti-Mullerian hormone receptor type 2 | Forward | TGCCACTACTTGACTCAGTACAC | NM_030998 |
|  |  | Reverse | GTGAGCGATACCTGGTTTGTACT |  |
| *Cyp19a1* | cytochrome P450 family 19 subfamily A member 1 | Forward | AAACTACTGCTTTGCGTGCTAAC | NM_017085 |
|  |  | Reverse | CAGGACCTGGTATGGAAGATGAG |  |
| *Fshr* | follicle stimulating hormone receptor | Forward | GTGTTCTCCAACCTACCCAAGTT | NM_199237 |
|  |  | Reverse | GGCAACGATGTGGATGTTTATGT |  |

**Supplementary Table S2. Antibodies information.**

| **Antibody** | **Species** | **Vendor (City, State)** | **Dilution** | | | |
| --- | --- | --- | --- | --- | --- | --- |
|  |  |  | **IF** | **FC** | **WB** | **IHC** |
| OCT4 | Mouse | Santa Cruz Biotechnology (Texas, USA) | 1:200 | ND | 1:1000 | ND |
| FOXL2 | Rabbit | ABclonal Biotechnology (Wuhan, China) | 1:200 | ND | 1:1000 | ND |
| FSHR | Rabbit | ABclonal Biotechnology (Wuhan, China) | 1:200 | ND | 1:1000 | ND |
| AMHR2 | Mouse | Santa Cruz Biotechnology (Texas, USA) | 1:200 | ND | ND | ND |
| CYP19A1 | Mouse | Santa Cruz Biotechnology (Texas, USA) | 1:200 | ND | ND | ND |
| CD90-FITC | Mouse | BioLegend (CA, USA) | 1:200 | 1:200 | ND | ND |
| CD45-PE | Mouse | BioLegend (CA, USA) | 1:200 | 1:200 | ND | ND |
| CD29-PE | Armenian Hamster | BioLegend (CA, USA) | 1:200 | 1:200 | ND | ND |
| CD34 | Mouse | Santa Cruz Biotechnology (Texas, USA) | 1:200 | 1:200 | ND | ND |
| CD44 | Mouse | Santa Cruz Biotechnology (Texas, USA) | 1:200 | 1:200 | ND | ND |
| CD63 | Rabbit | BioLegend (CA, USA) | ND | ND | 1:1000 | ND |
| TSG101 | Rabbit | BioLegend (CA, USA) | ND | ND | 1:1000 | ND |
| CD81 | Rabbit | BioLegend (CA, USA) | ND | ND | 1:1000 | ND |
| Calnexin | Rabbit | BioLegend (CA, USA) | ND | ND | 1:1000 | ND |
| phospho-FOXO3A | Rabbit | ABclonal Biotechnology (Wuhan, China) | ND | ND | ND | 1:200 |
| FOXO3A | Rabbit | ABclonal Biotechnology (Wuhan, China) | ND | ND | ND | 1:200 |
| HRP-conjugated goat anti-rabbit | Rabbit | MultiSciences(HangZhou, China) | ND | ND | 1:2000 | 1:200 |
| HRP-conjugated goat anti-mouse | Mouse | MultiSciences (HangZhou, China) | ND | ND | 1:2000 | ND |
| Alexa Fluor 594 Affinipure Donkey Anti-Rabbit IgG(H+L) | Rabbit | Yeasen Biotechnology (Shanghai, China) | 1:200 | 1;200 | ND | ND |
| Alexa Fluor 488 Affinipure Donkey Anti-Rabbit IgG(H+L) | Rabbit | Yeasen Biotechnology (Shanghai, China) | 1: 200 | 1: 200 | ND | ND |
| Alexa Fluor 594 Rabbit Anti-Mouse IgG(H+L) | Mouse | Yeasen Biotechnology (Shanghai, China) | 1:200 | 1:200 | ND | ND |
| Alexa Fluor 488 AffiniPure Rabbit Anti-Mouse IgG(H+L) | Mouse | Yeasen Biotechnology (Shanghai, China) | 1:200 | 1:200 | ND | ND |

ND = not detected; IF= Immunofluorescence; FC= Flowcytometry.


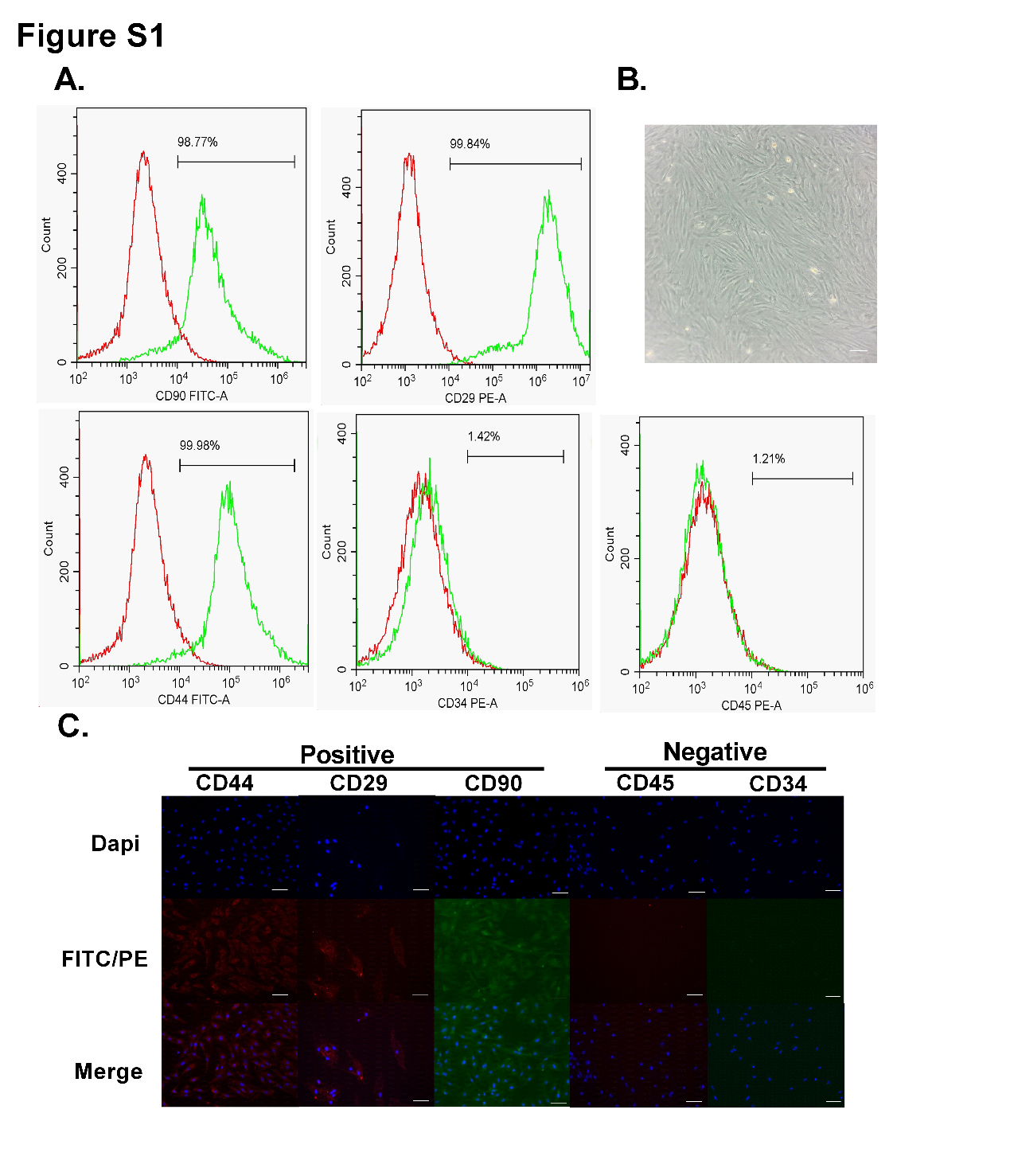


Figure S1. Isolation and identification of rat UCMSCs. A. rUCMSCs strongly positively (more than 95%) expressed CD44, CD90 and CD29 and negatively (less than 5%) expressed the negative cocktail, which included CD45 and CD34. B. The isolated rUCMSCs exhibited a typical fibroblast-like morphology. Scale bar: 100 µm. C. Immunofluorescence images of rUCMSC surface marker proteins. CD44, CD29, and CD90: umbilical cord mesenchymal stem cell markers; CD45 and CD34: markers of leukocytes and hematopoietic stem cells; nuclei were stained with DAPI (blue). Scale bar: 100 µm.


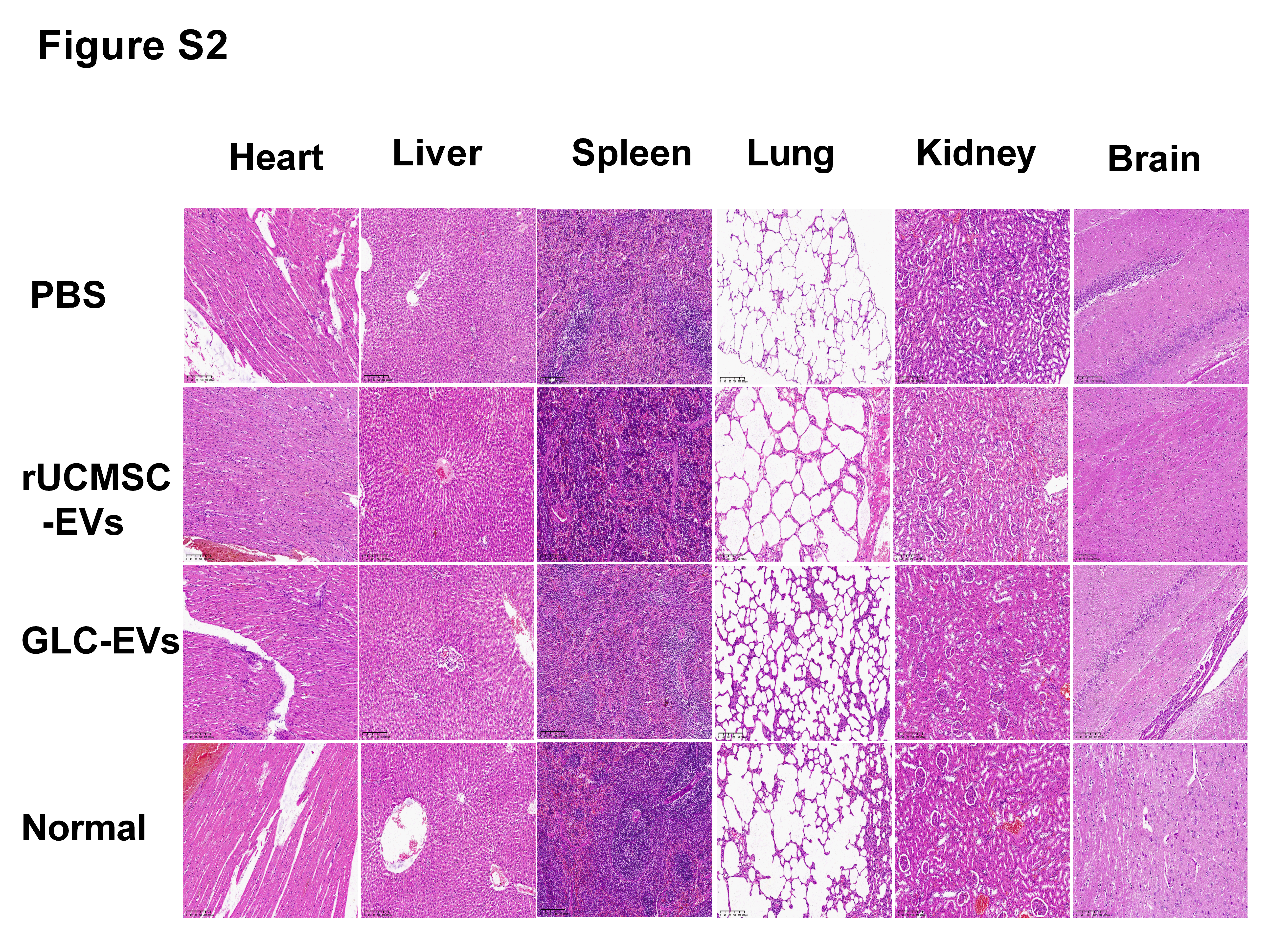


Figure S2. Histological analysis of other important organs. Scale bar: 200 µm（Scale bars are provided in the bottom-left corner of the images）.
